# The Positive Correlation of the Enhanced Immune Response to PCV2 Subunit Vaccine by Conjugation of Chitosan Oligosaccharide with the Deacetylation Degree

**DOI:** 10.3390/md15080236

**Published:** 2017-07-26

**Authors:** Guiqiang Zhang, Gong Cheng, Peiyuan Jia, Siming Jiao, Cui Feng, Tao Hu, Hongtao Liu, Yuguang Du

**Affiliations:** 1 University of Chinese Academy of Sciences, Beijing 100049, China; gqzhang@ipe.ac.cn; 2 Key Laboratory of Biopharmaceutical Production & Formulation Engineering, PLA and State Key Laboratory of Biochemical Engineering, Institute of Process Engineering, Chinese Academy of Sciences, Beijing 100190, China; gcheng@ipe.ac.cn (G.C.); pyjia@ipe.ac.cn (P.J.); smjiao@ipe.ac.cn (S.J.); cfeng@ipe.ac.cn (C.F.)

**Keywords:** chitosan oligosaccharide, deacetylation degree, adjuvant, porcine circovirus type 2 (PCV2), conjugation

## Abstract

Chitosan oligosaccharides (COS), the degraded products of chitosan, have been demonstrated to have versatile biological functions. In primary studies, it has displayed significant adjuvant effects when mixed with other vaccines. In this study, chitosan oligosaccharides with different deacetylation degrees were prepared and conjugated to porcine circovirus type 2 (PCV2) subunit vaccine to enhance its immunogenicity. The vaccine conjugates were designed by the covalent linkage of COSs to PCV2 molecules and administered to BALB/c mice three times at two-week intervals. The results indicate that, as compared to the PCV2 group, COS–PCV2 conjugates remarkably enhanced both humoral and cellular immunity against PCV2 by promoting lymphocyte proliferation and initiating a mixed T-helper 1 (Th1)/T-helper 2 (Th2) response, including raised levels of PCV2-specific antibodies and an increased production of inflammatory cytokines. Noticeably, with the increasing deacetylation degree, the stronger immune responses to PCV2 were observed in the groups with COS-PCV2 vaccination. In comparison with NACOS (chitin oligosaccharides)–PCV2 and LCOS (chitosan oligosaccharides with low deacetylation degree)–PCV2, HCOS (chitosan oligosaccharides with high deacetylation degree)–PCV2 showed the highest adjuvant effect, even comparable to that of PCV2/ISA206 (a commercialized adjuvant) group. In summary, COS conjugation might be a viable strategy to enhance the immune response to PCV2 subunit vaccine, and the adjuvant effect was positively correlated with the deacetylation degree of COS.

## 1. Introduction

Chitosan, the *N*-deacetylated derivative of chitin, is an important functional biomaterial because of its biological activities. Chitosan has been extensively studied as a vaccine adjuvant, mainly for use under oral and nasal administration. Chitosan oligosaccharides (COS), the degraded products of chitosan, have been demonstrated to have versatile biological functions such as being anti-tumor, immuno-stimulating, anti-inflammatory, anti-oxidation and working against bacterial infection [1,2]. Further to this, COS was shown to be well soluble, biocompatible, biodegradable, non-toxic and non-allergenic [3,4]. Recently, it was reported that the administration of a physical mixture of COS and H5N1 influenza vaccine or *Vibrio anguillarum* vaccine significantly activated the immune response of the host and was beneficial to the inhibition of pathogens [5,6]. In our primary studies, COS also showed significant adjuvant effects by physically mixing with porcine reproductive and respiratory syndrome virus vaccine (submitted) or covalently linking to PCV2 vaccine [7]. In addition, it was reported that the biological activities of chitosan were related to their size and deacetylation degrees [8,9], as was also confirmed by our study [10]. Based on the above, it is of interest to explore the effect of deacetylation degree (DD) on the adjuvant potency of COS applied to vaccines such as PCV2.

Porcine circovirus type 2 (PCV2) is the main cause of post-weaning multi-systemic wasting syndrome (PMWS) and other PCV-associated diseases (PCVD) [11], which was estimated to have caused around £88 million in losses per year during the epidemic period [12]. To control the PCV2 infection, vaccination has been regarded as one of the most effective tools in swine herds. Currently, the available commercial vaccines comprise the entire inactivated PCV2 virus, the inactivated chimeric PCV1-2 and the subunit of open reading frame 2 [13]. Among them, the PCV2 subunit vaccines are of great interest in modern immunotherapy, as they are safe, easy to produce and well defined. On the other hand, because of a lack of the necessary co-stimulatory factors, PCV2 subunit vaccines usually require the use of an adjuvant for optimal efficacy. Unfortunately, the adverse side effects of commercial adjuvants [14], such as toxicities and injection site necrosis, have been a bottleneck impeding their safe application [15]. Thus, new adjuvants such as COS with a low toxicity have been of particular interest in meeting the demands of PCV2 inoculation.

Generally, the adjuvant was delivered by physically mixing it with the antigen to activate antigen presenting cells (APCs) but not directly present the antigen and induce the potential autoimmune responses [16,17]. In addition, in most cases, a high dose of adjuvant is needed to maintain the satisfactory immune-enhancing responses, even though this will lead to unwanted side effects. To ensure the strong immunogenicity of the adjuvant while avoiding its possible toxicity, an antigen-adjuvant conjugate was designed by the covalent linkage of antigen molecules and the adjuvant. By this conjugation, both the antigen and adjuvant can reach the APC simultaneously and initiate a much higher immune reaction as compared to that achieved by the simple mixture of antigen plus adjuvant [18]. Especially, such an antigen–adjuvant conjugate can minimize the side effects of an adjuvant by decreasing the given dosage. Since this new strategy was put forward, it has been widely accepted and applied to the developing of adjuvants. 

In present study, three COS–PCV2 conjugates were developed by the covalent linkage of COSs with different DD to PCV2 molecules. The immunological property of each conjugate was measured and the adjuvant potencies of these COS-PCV2 conjugates were assessed, including an analysis on serum antibody responses, the detection of cytokine secretion by spleen lymphocytes and the evaluation of lymphocyte proliferation. Moreover, MONTANIDE™ ISA206, a commercial adjuvant, was compared as the positive control.

## 2. Results

### 2.1. Purification and Quantitative Analysis of Conjugates

**Figure 1:**
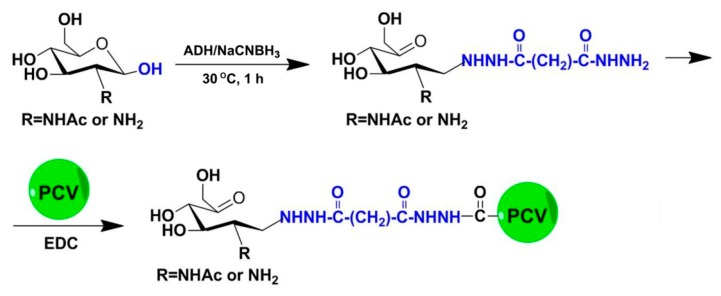
Reaction scheme of the chitosan oligosaccharides–porcine circovirus type 2 (COS-PCV2) conjugate synthesis.

Chitosan oligosaccharides with a high deacetylation degree (HCOS) were prepared from enzymatic hydrolysis as described previously [19]. In brief, chitosan was dissolved in an acetate buffer, then the pH of the solution was adjusted to 6. The chitosanase from *Streptomyces griseus* was added to a final concentration of 0.5 μg/mg chitosan, and the reactions were incubated with shaking (500 rpm) at 40 °C. The reaction was stopped by decreasing the pH to 2.5 with HCl. Then, HCOS was acetylated to prepare chitin oligosaccharides (NACOS) and chitosan oligosaccharides with a low deacetylation degree (LCOS) according to the method reported by Li’s group [20]. The component information of three COSs were determined by TOF-MS, and average molecular weights were determined to be below 1 kDa by HPLC using Evaporative Light-scattering Detector (ELSD) detection on a TSKgel G4000PWXL (Tosoh Corporation, Tokyo, Japan) column (7.8 mm × 300 mm), with 1 k, 5 k and 25 kDa dextran as standards (Supplementary Figures S1–S3). Their deacetylation degrees were also separately identified to be 4%, 60% and 88% by ^1^H-NMR spectroscopy [21] (Supplementary Figure S4). To prepare the conjugates, the reducing ends of COSs were derived with adipic acid dihydrazide by using reductive amination as previously reported [22], followed by conjugation to PCV (Figure 1).

**Figure 2:**
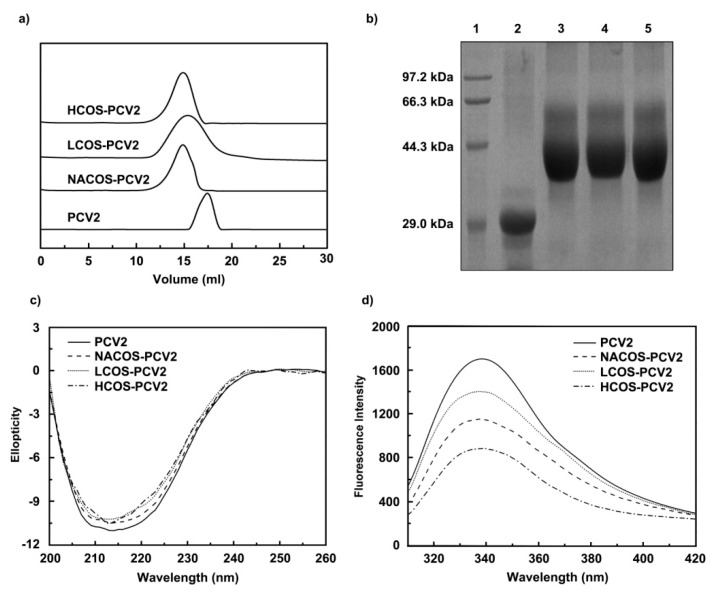

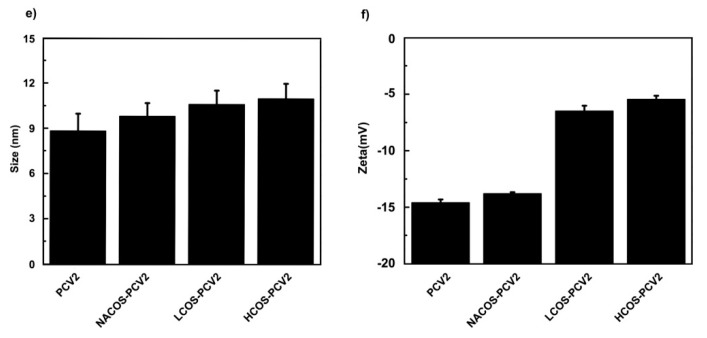
Chromatographic analysis, purification and physical characterization of COS-PCV2 conjugates. (**a**) The purification of COS-PCV2 conjugates preformed on a Superdex 75 column (2.6 cm × 70 cm); (**b**) The chromatography of free PCV2 and COS-PCV2 conjugates by SDS-PAGE (12%) assay. Lane 1: Marker; Lane 2: PCV2; Lane 3: chitin oligosaccharides (NACOS)-PCV2; Lane 4: chitosan oligosaccharides with a low deacetylation degree (LCOS)-PCV2; Lane 5: chitosan oligosaccharides with a high deacetylation degree (HCOS)-PCV2. (**c**–**f**) Physical characterization of free PCV2 and COS-PCV2 conjugates detected by CD spectroscopy assay (**c**); fluorescence analysis (**d**) and dynamic light scattering (DLS) assay (**e**–**f**).

Next, a Superdex 75 column (2.6 cm × 70 cm) was used to purify the free and conjugated PCV2 from the reaction mixture, and the corresponding fractions were collected. After purification, the fraction samples were assayed on an analytical Superdex 75 column (1.0 cm × 30 cm). As shown in Figure 2a, free PCV2 was eluted as a single peak at 17.2 mL, while all three peaks of COS-PCV2 conjugates shifted toward the left, and the elution volumes were 14.9 mL for NACOS-PCV2, 15.0 mL for HCOS-PCV2 and 15.3 mL for LCOS-PCV2, respectively. Furthermore, the purified PCV2 conjugates were analyzed by SDS-polyacrylamide gel electrophoresis (PAGE), showing strong smear bands with a higher mass compared to that of free PCV2 (Figure 2b). In addition, a higher carbohydrate/protein ratio (*w*/*w*) was observed for HCOS-PCV2 (0.138) than those of NACOS-PCV2 (0.126) and LCOS-PCV2 (0.127), indicating a more efficient conjugation. A low residual level of unconjugated carbohydrate for each reaction mixture was detected after the purification process, ranging from 2.0 to 4.5%.

### 2.2. Physicochemical Characterization

#### 2.2.1. Circular Dichroism (CD) Spectroscopy Assay

CD analysis was used to investigate the secondary structure of free or conjugated PCV2 vaccines. As compared to free PCV2, the PCV2 conjugates displayed lower ellipticity values at 215 nm, indicating the slightly decreased α-helix content in PCV2 by COS conjugation (Figure 2c).

#### 2.2.2. Fluorescence Measurement

Intrinsic fluorescence was measured to detect the conformational changes in PCV2 after COS conjugation, as revealed by the changes of Trp, Tyr and Phe residues exposed to the solvent. In contrast to free PCV2, the PCV2 conjugates showed a noticeable decrease in emission fluorescence intensity without a noticeable shift at the maximum wavelength (Figure 2d). This may be due to the presence of carbonyl moieties in conjugates, which could quench the florescence intensity of PCV2.

#### 2.2.3. Dynamic Light Scattering (DLS) Analysis

As measured by DLS, free PCV2 exhibited a molecular radius of 8.83 nm, which was lower than those of NACOS-PCV2 (9.82 nm), LCOS-PCV2 (10.62 nm) and HCOS-PCV2 (11.01 nm) (Figure 2e). From the result, it can be presumed that the molecular volume of PCV2 might be increased by COS conjugation.

The charges of free or conjugated PCV2 were also analyzed by DLS. As shown in Figure 2f, free PCV2 showed a zeta potential of −14.6 mV, lower than that NACOS-PCV2 (−13.8 mV). Remarkably, significantly higher zeta potentials were observed for LCOS-PCV2 (−6.49 mV) and HCOS-PCV2 (−5.45 mV). It is suggested that the zeta potential of PCV2 with a negative charge was evidently increased by COS conjugation.

### 2.3. PCV2-Specific Antibody Production

**Figure 3:**
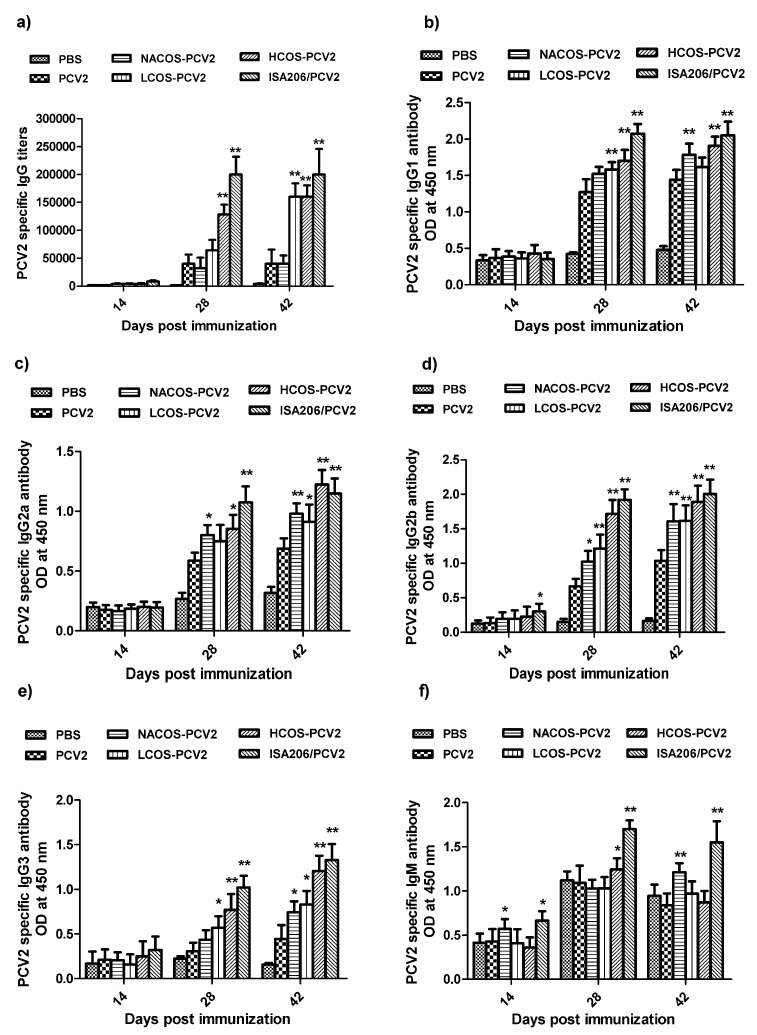
Effect of COS conjugation on PCV2-specific antibody production of IgG, IgG isotypes and IgM in mouse serum. Mice were intramuscularly injected with PBS, PCV2, NACOS-PCV2, LCOS-PCV2, HCOS-PCV2 or ISA206/PCV2 mixture on day 0, 14 and 28. The blood samples were collected at day 14, 28 and 42 dpi. The antibody titers of IgG (**a**); IgG1 (**b**); IgG2a (**c**); IgG2b (**d**); IgG3 (**e**) and IgM (**f**) were determined by ELISA, respectively. Data are represented as means ± SD (*n* = 6) of duplicate wells. * *p* < 0.05 or ** *p* < 0.01, compared to the PCV2 group.

PCV2-specific antibody levels of IgG, IgG isotypes and IgM in mouse serum were assessed by ELISA. As shown in Figure 3a–f, relatively low antibody levels were produced over the whole vaccination period in groups administered by PBS and PCV2. In contrast, the conjugation of PCV2 with NACOS, LCOS and HCOS significantly increased PCV2-specific antibody titers, including IgG, IgG1, IgG2a, IgG2b and IgG3 (*p* < 0.05 or 0.01, vs. the PCV2 group). In particular, about a four-fold increase in the PCV2-specific IgG antibody was detected in the groups with LCOS-PCV2 or HCOS-PCV2 administration at the third immunization, slightly lower than that of the ISA206/PCV2 group.

As an indicator to analyze the Th1 or Th2-biased immune responses to vaccination, the IgG2a/IgG1 ratio was also obtained from each group after PCV2 immunization (Supplementary Table S1). In comparison with the PCV2 group, the IgG2a/IgG1 ratio of groups administered with COS conjugates was significantly increased after the third immunization (*p* < 0.05 or 0.01), and HCOS-PCV2 group displayed the highest IgG2a/IgG1 value. 

### 2.4. Lymphocyte Proliferation Assay

**Figure 4:**
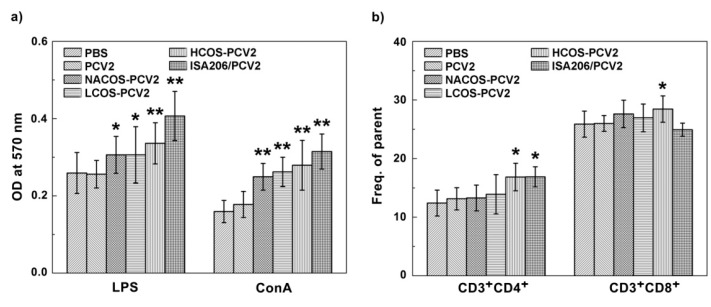
Effect of COS conjugation on lymphocyte proliferation in PCV2-vaccinated mice. On day 14, after the third immunization, mice were euthanized and splenic lymphocytes were prepared. After treatment with ConA (2 µg/mL) or LPS (10 µg/mL) for 48 h, the lymphocyte proliferation was analyzed by MTT assay (**a**); And the CD3^+^CD4^+^ and CD3^+^CD8^+^ cell populations were determined by flow cytometry analysis (**b**). Data are represented as the means ± SD (*n* = 6) of duplicate wells. * *p* < 0.05 or ** *p* < 0.01, compared to the PCV2 group.

T and B-lymphocyte proliferation can be separately activated by ConA or LPS. To determine whether the lymphocyte proliferation response to PCV2 vaccination was boosted by COS conjugation, the primary lymphocytes from mouse spleens were isolated at 42 dpi and the proliferation assay was assessed by Methylthiazolyldiphenyl-tetrazolium bromide (MTT) analysis. As indicated in Figure 4a, the COS conjugation with PCV2 led to a significant increase in B-cell proliferation after LPS (10 μg/mL) stimulation for 48 h compared with that of the PCV2 group (*p* < 0.05 or 0.01). The HCOS-PCV2 group showed the highest proliferation activity, but less than that of the ISA206/PCV2 group. Similar results were also observed in T-cell proliferation test after the lymphocytes were stimulated by ConA (2 μg/mL) for 48 h. 

### 2.5. Subpopulation Analysis of CD3^+^ T Cells

CD4^+^ T cells represent a major T cell population and are well known as T helper cells, which are mainly associated with inflammatory cytokine production by Th1 immune response and increased antibody secretion by Th2 activation [23]. On the other hand, CD8^+^ T cells play an important role in immune protection against viral infections [23]. To further determine the effect of COS conjugation on T cell activation, the subpopulations of both CD3^+^CD4^+^ T cell and CD3^+^CD8^+^ T cell from mouse spleens of experimental groups were quantified by flow cytometry analysis. As indicated in Figure 4b and Supplemental Figures S5 and S6, NACOS-PCV2 and LCOS-PCV2 slightly, but not statistically, increased either CD4^+^ or CD8^+^ lymphocytes compared to that by PCV2 vaccination alone. By contrast, both T cell subpopulations were significantly elevated by HCOS-PCV2 vaccination as compared to the PCV2 group (*p* < 0.05). In addition, the CD4^+^ lymphocyte population from the ISA206/PCV2 group was also increased in comparison with that of the PCV2 group (*p* < 0.05). 

### 2.6. Cytokine Assays

The activation of immune responses was usually characterized by the up-regulation of interleukin (IL)-2 and interferon (IFN)-γ (typical of Th1 response), the increase of IL-4 (typical of Th2 response) or the increment of tumor necrosis factor (TNF)-α (typical of cytotoxic reaction) [24]. To monitor the effect of COS conjugation on PCV2-specific cytokine secretion, the primary lymphocytes were isolated from mouse spleens at 42 dpi. Cells from different groups were treated with ConA (2 μg/mL) or LPS (10 μg/mL) for 48 h. After that, the levels of above mentioned cytokines in culture supernatant were assayed by ELISA.

**Figure 5:**
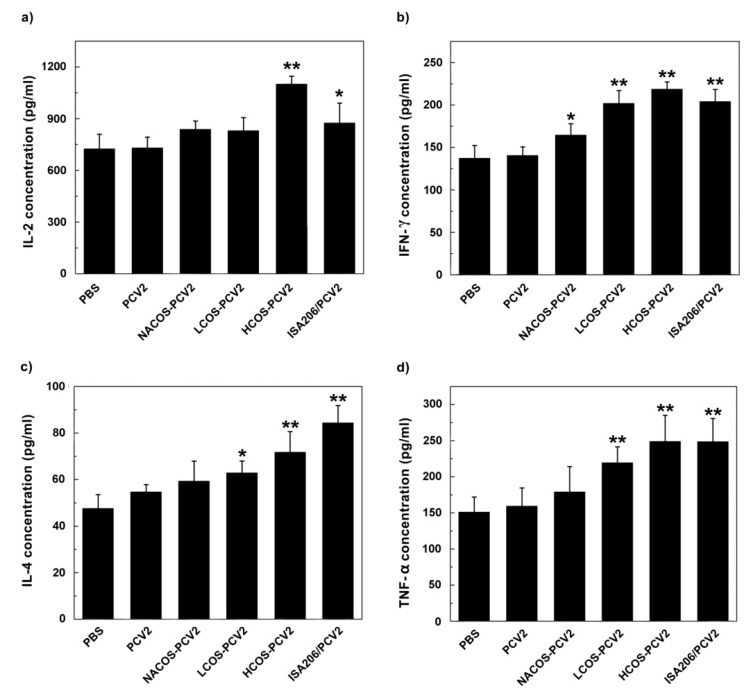
Effect of COS conjugation on the production of IL-2 (**a**); IFN-γ (**b**); IL-4 (**c**) and TNF-α (**d**) secreted by spleen lymphocytes from immunized mice. On day 14, after the third immunization, mice were euthanized and splenic lymphocytes were prepared. After cells were treated with Con A (2 μg/mL, for IL-2, IFN-γ and IL-4 assay) or LPS (10 μg/mL, for TNF-α assay) for 48 h, the culture supernatant was collected for cytokine detection by ELISA. Data are represented as the means ± SD (*n* = 6) of duplicate wells. * *p* < 0.05 or ** *p* < 0.01, compared to the PCV2 group.

As shown in Figure 5, the levels of cytokines above four from groups with LCOS-PCV2 or HCOS-PCV2 vaccination were statistically higher than that of the PCV2 group (*p* < 0.05 or 0.01). Notably, the stimulant effect by HCOS-PCV2 on the secretion of IL-2 and IFN-γ were even stronger than that by ISA206/PCV2 (*p* < 0.05). The result indicates that COS conjugation can promote cytokine secretion initiated by PCV2, which is positively correlated with the deacetylation degree.

### 2.7. Viscera Index and Histopathological Assay

**Figure 6:**
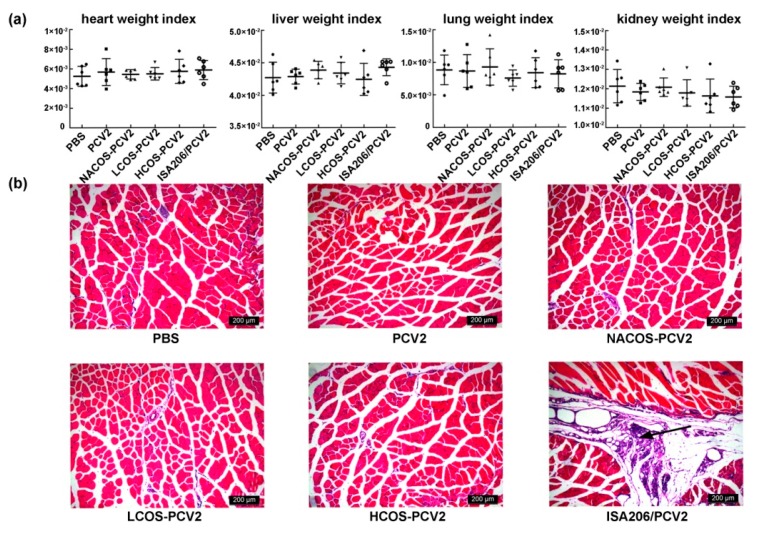
Effect of COS conjugation on the viscera index and injection site in PCV2-vaccinated mice. At 42 dpi, heart, lung, liver or kidneys of the mice were immediately separated and viscera indexes for them were calculated (**a**), and the tibialis muscle tissues near injection sites were collected for pathological examination by hematoxylin and eosin (HE)-staining (**b**). Solid arrows represented the inflammatory cell infiltration. Magnification: 100×.

We observed mice behaviour, autonomic activities, ingestion, drinking, hairs, faeces and urine daily. No lethality or clinical signs were observed for all vaccinated groups over the entire immunization period. However, remarkable lesions were observed at the injection sites of the mice from the ISA206/PCV2 group after each immunization, whereas no pathological signs were found in other groups. As indicated in Figure 6a, the viscera index of heart, lung and kidney show no significant difference between each other. Compared to the PBS group, the liver index of mice in NACOS-PCV2 and ISA206/PCV2 group showed a slight, but not statistically significant, increase (*p* > 0.05).

The histopathological examination of tibialis muscle sections near the injection sites shows that the mice with ISA206/PCV2 immunization displayed severe infectious symptoms characterized by inflammatory cell infiltration, as the arrow indicates in Figure 6b, while no microscopic lesions were observed in the groups administered with PBS, PCV2 alone or PCV2-COS conjugates. Almost all commercial adjuvants were found to cause side effects to certain extents, including injection site lesions, ulcerations and other systemic responses such as fever and lethargy. In addition, some of the above adverse symptoms can even be detected in animal products that might cause potential food safety issues. In line with that reported above, our study shows that no toxicity or other side effects were observed after the administration of COS adjuvants at injection sites of mice or in major metabolic organs (liver and kidney), while the oil emulsion, i.e., ISA206, led to severe histopathological changes [7]. Based on the above, it can be concluded that the COS–PCV2 conjugates had no side effects on the mice.

## 3. Discussion

To date, traditional adjuvants such as oil-in-water or water-in-oil emulsion are still widely applied to livestock vaccination due to their strong immune efficacy, even though they have been proven to cause severe toxic reactions or even produce a potential health risk for human beings [25,26]. It seems that the livestock industry has no better choice but to continue to use oil emulsion as the major adjuvant to PCV2 vaccination until totally new adjuvants are developed [27]. As an adjuvant candidate, COS has showed excellent immune-enhancing activity in vitro and in vivo. In this study, we further investigated the effect of deacetylation degree on the adjuvant potency of COS targeting the PCV2 subunit vaccine. For the first time, we have found that COS with a high deacetylation degree displayed a better adjuvant effect on PCV2 subunit vaccine. Notably, it showed no infection or pathological signs at the injection sites of the mice.

It was reported that PCV2-specifc antibodies are associated with protection against virus infection, as evidenced by the contribution of reduced antibodies to the development of PCVD [28]. In this study, three antigen-adjuvant conjugates were designed by the conjugation of COSs with different degradation degrees to PCV2 subunit vaccine, and the immunogenicity of each COS-PCV2 conjugate was evaluated using BALB/c mice. From the results, the PCV2-specific antibody levels elicited by COS-PCV2 conjugates were shown to be significantly higher than that of the purely PCV2 group, suggesting an enhanced humoral immune response to PCV2 vaccine by COS conjugation. Among three conjugates, HCOS-PCV2 presented the strongest stimulant effect on antibody production compared to NACOS-PCV2 and LCOS-PCV2. Considering that the only difference among the three COSs is their variation of deacetylation degrees, this result implies a key role of COS structure to their biological activities. In addition, all three COS-PCV2 conjugates showed an increase in their IgG2a/IgG1 ratios compared with the PCV2 vaccine alone (Supplementary Table S1). It is thus plausible that COS conjugation may up-regulate the Th1-biased immune response.

CD4^+^ T cells represent the major T cell population and are mainly associated with Th1 and Th2 immune responses via cytokine production and antibody secretion [29], while CD8^+^ T cells play an important role in protection against viral infections [23]. In the present study, COS conjugation not only increased the proliferation of spleen lymphocytes including CD4^+^ T cells, CD8^+^ T cells and B cells, but markedly raised the levels of inflammatory cytokines including IL-2, IL-4, IFN-γ and TNF-α. As IL-2 is the central regulator of Th1 response and IL-4 has been proved to enhance Th2 response, our results point toward a mixed Th1/Th2 response in groups with COS-PCV2 conjugates. In addition, HCOS-PCV2 vaccination dramatically elevated the level of CD8^+^ T cells compared with the PCV2 alone group (Figure 4b), alluding to the activation of cytotoxic response to PCV2. Once again, HCOS exhibited a higher adjuvant efficacy than that by NACOS or LCOS.

There are several factors that may affect the immune response to conjugates, such as adjuvant loading, conjugation method, immunization protocol, and antigen structure [30]. For three COS-PCV2 conjugates, their adjuvant loadings, conjugation methods and immunization protocols were the same. We thus presume that it is the variation of chemical structure that determines the differences in the immunological properties of COSs. All three COSs are composed of D-glucosamine with a similar polymerization degree, but their degradation degrees vary greatly (4% for NACOS, 60% for LCOS, 88% for HCOS). Previous studies have demonstrated that the substituting NH_2_ group could form the ammonium group NH_3_^+^ by absorbing hydrion from the solution, and this positively charged character makes it easy to bind or react with other molecules [31]. It is consistent with our results that the zeta potentials were evidently increased by COS conjugation (Figure 2f). Moreover, such positive charges can promote cellular uptake and change intracellular trafficking [32]. Another mechanism to explain the potency of COS-PCV2 conjugates may related to the activation of macrophages and dendritic cells via the mannose receptor or the Toll-like receptor 4 (TLR4) by COS [33,34]. Our results are in line with those reported by Blander et al. [35] and Khan et al. [36], who showed that the enhancement of cellular immunity is associated with the antigen linkage to ligands targeting Toll-like receptors (TLRs). To explore the exact mechanism about our interesting findings, a good deal of experiments will be performed in the future work.

## 4. Materials and Methods

### 4.1. Reagents

The chitosan samples were purchased from Sigma-Aldrich China Inc. (Shanghai, China). The molecular weight was 1.6 × 10^5^ and the degree of deacetylation was above 75%. The PCV2 subunit vaccine was obtained from Pulike biological engineering, Inc. (Luoyang, China). Adipic dihydrazide (ADH), 1-(3-dimethylaminopropyl)-3-ethylcarbodimide (EDC), sodium cyanoborohydride (NaCNBH_3_), mouse monoclonal antibody isotyping reagents, horse radish peroxidase (HRP)-conjugated goat anti-mouse IgG polyclonal antibody and 3,3′,5,5′-tetramethylbenzidine (TMB) were purchased from Sigma-Aldrich China Inc. Mouse TNF-α, mouse IL-2, IL-4 and IFN-γ platinum ELISAs were purchased from eBioscience (San Diago, CA, USA).

### 4.2. Preparation and Purification of COS-PCV2 Conjugates

COSs were solubilized in AcONa (100 mM, pH 4.5) at a concentration of 40 mg/mL. ADH and NaBH_3_CN were separately added as solids with a ratio of 1.2:1 by weight to each COS. The solution was mixed at 30 °C for 1 h (Figure 1). After this, the reaction mixture was desalted on a Sephadex™ G-25 Superfine desalting column (GE Healthcare, Fairfield, CT, USA).

For the PCV2 vaccine conjugation, the COS–ADH was allowed to react with PCV2 in the presence of EDC in 100 mM MES buffer (pH 4.5) at room temperature overnight (Figure 1). The excessive EDC was removed by extensive dialysis against 0.15 M NaCl solution, using a Slide-A-Lyzer dialysis cassette (MWCO 3 kDa, Thermo Scientific, Waltham, MA, USA) at 4 °C.

A size exclusion chromatograph (SEC) based on a Superdex 75 column (2.6 cm × 70 cm, GE Healthcare, Fairfield, CT, USA) was used to purify the COS–PCV2 conjugates from reaction mixture. The column was equilibrated and eluted by phosphate buffer saline (PBS, 20 mM, pH 7.4) at a flow rate of 3.0 mL/min. The fractions corresponding to the COS–PCV2 conjugates were pooled and concentrated using an Amicon membrane with 3 kDa cutoff at 4 °C. 

### 4.3. Characterization Analysis on COS–PCV2 Conjugates

#### 4.3.1. Quantitative Assay

The total carbohydrate content of COS–PCV2 conjugates was measured using the phenol-sulphuric acid colorimetric method as described [37]. The quantification of the unconjugated carbohydrate was performed by an ethanol precipitation assay [38]. The protein content was measured using the micro bicinchoninic acid method (Solarbio, Beijing, China) to determine the ratio of carbohydrate to protein (*w*/*w*) of the PCV2 conjugates.

#### 4.3.2. CD Spectroscopy Assay

The CD measurement of free or conjugated PCV2 was carried out on a Jasco-810 spectropolarimeter (Jasco, Tokyo, Japan) using a cuvette with 0.2 cm pathlength [39]. All samples were at a protein concentration of 0.1 mg/mL in PBS, and the PBS solution baseline was subtracted from the experimental spectra for corrections.

#### 4.3.3. DLS Analysis

The DLS analysis was performed to measure the molecular radii and zeta potential of free or conjugated PCV2 using a Malvern Zetasizer Nano ZS instrument (Malvern Instruments Ltd., Malvern Worcestershire, UK) at 25 °C [40]. The samples were at a protein concentration of 1.0 mg/mL in PBS. All samples were centrifuged at 12,000× *g* for 10 min before analysis.

#### 4.3.4. Fluorescence Measurement

The intrinsic fluorescence was measured using Hitachi F-4500 Fluorescence spectropolarimeter (Hitachi, Tokyo, Japan) with a 1.0 cm pathlength cuvette [41]. The measurement was performed at a protein concentration of 0.1 mg/mL in PBS at room temperature. The emission spectra (300–450 nm) were excited at 280 nm with a slit width of 5.0 nm. The PBS solution baseline was subtracted from the experimental spectra for correction.

### 4.4. Animal Immunization

The BALB/c mice aged 4–6 weeks (15–20 g) were randomly divided into six groups (*n* = 6); i.e., PBS group, PCV2 group, NACOS-PCV2 group, LCOS-PCV2 group, HCOS-PCV2 group and ISA206/PCV2 mixture group. For each group, the mice were intramuscularly injected on days 0, 14 and 28 with 0.1 mL of PBS, free PCV2, COS-PCV2 or ISA206/PCV2 mixture at the protein concentration of 100 μg/mL. The blood samples were taken from the tail vein at 14, 28 and 42 days post primary immunization (dpi). After the centrifugation at 4 °C, the mouse sera were isolated and stored at −80 °C for further experiments.

All procedures for the animal experiment were approved by the Animal Ethical Experimentation Committee of Institute of Process Engineering, Chinese Academy of Sciences (Beijing, China), in accordance with the National Act on the Use of Experimental Animals (Beijing, China).

### 4.5. Detection of PCV2-Specific Antibodies

For the detection of PCV2-specific antibodies including IgG, IgG1, IgG2a, IgG2b, IgG3 and IgM, the serum samples were analyzed by modified enzyme-linked immunosorbent assay (ELISA). Briefly, 96-well microplates were coated with 5 μg/well PCV2 antigen in carbonate buffer (50 mM, pH 9.6) overnight at 4 °C. After this, the plates were washed three times with PBS containing 0.1% Tween 20 (PBST, 10 mM, pH 7.4) and blocked with 5% of skimmed milk in PBS for 1 h at 37 °C. After three washes, 100 μL of diluted serum samples were added into each well and incubated at 37 °C for 1 h followed by three washes. Then, the plates were incubated with 100 μL of HRP-GAM-IgG, IgG isotypes or IgM antibody at 37 °C for 1 h. After washing five times, 100 μL of TMB substrate was added and incubated at 37 °C in the darkness for 30 min, followed by quenching the reaction with 50 μL of H_2_SO_4_ (2 M). The optical density were read at 450 nm by using a Tecan Infinite M200 Pro microplate reader (Grodig, Austria).

### 4.6. Lymphocyte Proliferation Assay

At 42 dpi, lymphocytes were separated from mouse spleens using the lymphocyte separation medium, resuspended at 5 × 10^6^ cells/mL with RPMI-1640 complete medium containing 10% FBS, 100 units/mL penicillin and 100 µg/mL streptomycin. For the proliferation assay, 100 μL of lymphocyte suspension was seeded into each well and treated with lipopolysaccharides (LPS, 10 μg/mL) or concanavalin A (ConA, 2 μg/mL). After incubation at 37 °C with 5% CO_2_ for 48 h, the culture supernatant was removed and washed with PBS. Then, 100 µL of MTT (5 mg/mL) in complete medium was added and incubated for another 4 h. Followed by the removal of MTT, the colored formazan was dissolved in 100 μL of DMSO. The OD values were measured at 570 nm using a microplate reader as above, and the cell viability in each well was presented as percentage of the control level.

### 4.7. Flow Cytometry Analysis on T Cell Subpopulation

The flow cytometry analysis was performed to determine the subpopulation of spleen T-lymphocytes from immunized mice. Lymphocytes were prepared as before and stained with APC-labelled monoclonal antibody against CD3, FITC (fluorescein isothiocyanate)-conjugated monoclonal antibody against CD4 or PE-labelled monoclonal antibody against CD8 (1:1000 dilution) at 4 °C for 45 min. After incubation, the cells were washed with cold PBS for 3 times. After resuspension in PBS, the cells were subjected to flow cytometry.

### 4.8. Cytokine Assay

After the primary spleen lymphocytes were prepared as described above in 4.6, a lymphocyte suspension was seeded into each well and treated with lipopolysaccharides (LPS, 10 μg/mL) or concanavalin A (ConA, 2 μg/mL). After the incubation at 37 °C with 5% CO_2_ for 48 h, the culture supernatant in each well was collected to determine the levels of IL-2, IFN-γ, IL-4 and TNF-α by using commercial ELISA kits according to the protocols of the manufacturers.

### 4.9. Viscera Index and Histopathological Assay

At 42 dpi, the mice for each group were euthanized, and the heart, lung, liver or kidneys were immediately separated. The surrounding connective tissue and fat were excluded. The organs were dried of surface moisture using filter paper and weighed. Viscera indexes for the heart, lung, liver and kidney were calculated by the following formula: organ index = organ weight (mg)/body weight (g).

The tibialis muscle tissue samples at the injection sites were also collected and fixed in 4% neutral-buffered formalin solution. The tissue samples were then embedded in paraffin and cut into 4 μm thick slices. After hematoxylin and eosin (HE) staining, a histopathological analysis was performed and the microscopic images were photographed using a Leica DMI3000 B microscope (Wetzlar, Germany).

### 4.10. Statistical Analysis

Data are presented as means ± SD. A two-tailed Student’s *t* test was performed for the comparison between two groups and a one-way ANOVA for multiple group analysis. The *p* value < 0.05 or 0.01was considered be significant. All data were analyzed using SPSS 13.0 software (SPSS, Chicago, IL, USA).

## 5. Conclusions

In summary, COS conjugation markedly enhanced both humoral and cellular immune responses against PCV2 by promoting the spleen lymphocyte proliferation, which in turn skewed towards a mixed Th1/Th2 response, including elevated antibody production and raised cytokine secretion. Further, the adjuvant potencies by COSs are positively correlated with their deacetylation degree. The data presented here suggest that a high degree of deacetylation may benefit the immunogenicity of COS-PCV2 conjugates against the associated virus infection.

## Supplementary Materials

The following are available online at www.mdpi.com/1660-3397/15/8/236/s1, Figure S1: The structure of partially decaetylated COS; Figure S2: Determination of the component information of COSs by TOF-MS spectroscopy; Figure S3: The average molecular weights were below 1 kDa by HPLC using ELSD detection on a TSKgel G4000PWXL column (7.8 mm × 300 mm), with 1 k, 5 k and 25 kDa dextran as standards; Figure S4: Determination of the deacetylation degree (DD) of COSs by ^1^H-NMR spectroscopy; Figure S5: Effect of COS conjugation on CD3^+^CD4^+^ T cell population from mouse spleens by flow cytometry analysis; Figure S6: Effect of COS conjugation on CD3^+^CD8^+^ T cell population from mouse spleens by flow cytometry analysis; Table S1: Effect of COS conjugation on the ratio of PCV2-specific IgG2a to IgG1 in mouse serum after immunization.
