# Correction: Zhang, G.; Cheng, G.; Jia, P.; Jiao, S.; Feng, C.; Hu, T.; Liu, H.; Du, Y. The Positive Correlation of the Enhanced Immune Response to PCV2 Subunit Vaccine by Conjugation of Chitosan Oligosaccharide with the Deacetylation Degree. *Marine Drugs* 2017, *15*, 236

**DOI:** 10.3390/md15090292

**Published:** 2017-09-20

**Authors:** Guiqiang Zhang, Gong Cheng, Peiyuan Jia, Siming Jiao, Cui Feng, Tao Hu, Hongtao Liu, Yuguang Du

**Affiliations:** 1 University of Chinese Academy of Sciences, Beijing 100049, China; gqzhang@ipe.ac.cn; 2 Key Laboratory of Biopharmaceutical Production & Formulation Engineering, PLA and State Key Laboratory of Biochemical Engineering, Institute of Process Engineering, Chinese Academy of Sciences, Beijing 100190, China; gcheng@ipe.ac.cn (G.C.); pyjia@ipe.ac.cn (P.J.); smjiao@ipe.ac.cn (S.J.); cfeng@ipe.ac.cn (C.F.)

The authors wish to correct Figure 1 in this paper [1] to be as follows:

The authors wish to correct the order of references 9 and 10 in References Part of [1] to be as follows:

9. Wang, Z.; Zheng, L.; Yang, S.; Niu, R.; Chu, E.; Lin, X. *N*-acetylchitooligosaccharide is a potent angiogenic inhibitor both in vivo and in vitro. *Biochem. Biophys. Res. Commun.* **2007**, *357*, 26–31.
10. Wu, H.; Aam, B.B.; Wang, W.; Norberg, A.L.; Sørlie, M.; Eijsink, V.G.; Du, Y. Inhibition of angiogenesis by chitooligosaccharides with specific degrees of acetylation and polymerization. *Carbohydr. Polym.* **2012**, *89*, 511–518.

## Figures and Tables

**Figure 1:**
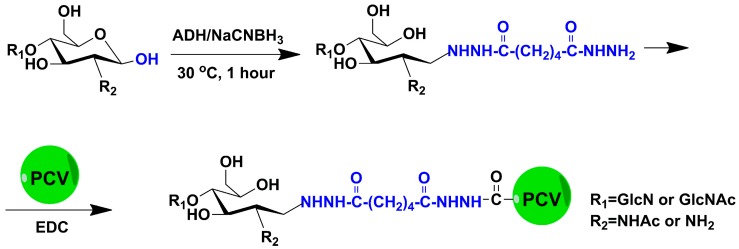
Reaction scheme of the chitosan oligosaccharides–porcine circovirus type 2 (COS-PCV2) conjugate synthesis.
