# Supplementary material for: The Positive Correlation of the Enhanced Immune Response to PCV2 Subunit Vaccine by Conjugation of Chitosan Oligosaccharide with the Deacetylation Degree
Source: Mar Drugs. 2017 Jul 26;15(8):236. doi: 10.3390/md15080236 (PMC5577591; doi:10.3390/md15080236)
Supplement: Supplementary file 1 [file marinedrugs-15-00236-s001.pdf]

Supplementary Information

Files in this data supplement

Supplementary Fig. 1

Supplementary Fig. 2

Supplementary Fig. 3

Supplementary Fig. 4

Supplementary Fig. 5

Supplementary Fig. 6

Supplementary Table 1

Fig.1. The structure of partially decaetylated COS. DD, a copolymer characterized by the average deacetylation degree.

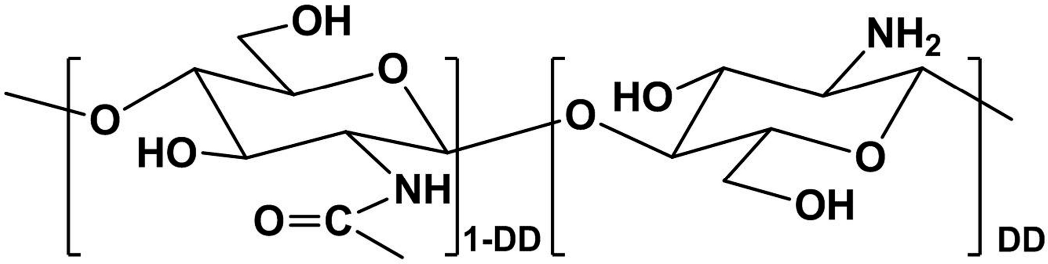

Fig. 2. Determination of the component information of COSs by TOF-MS spectroscopy.

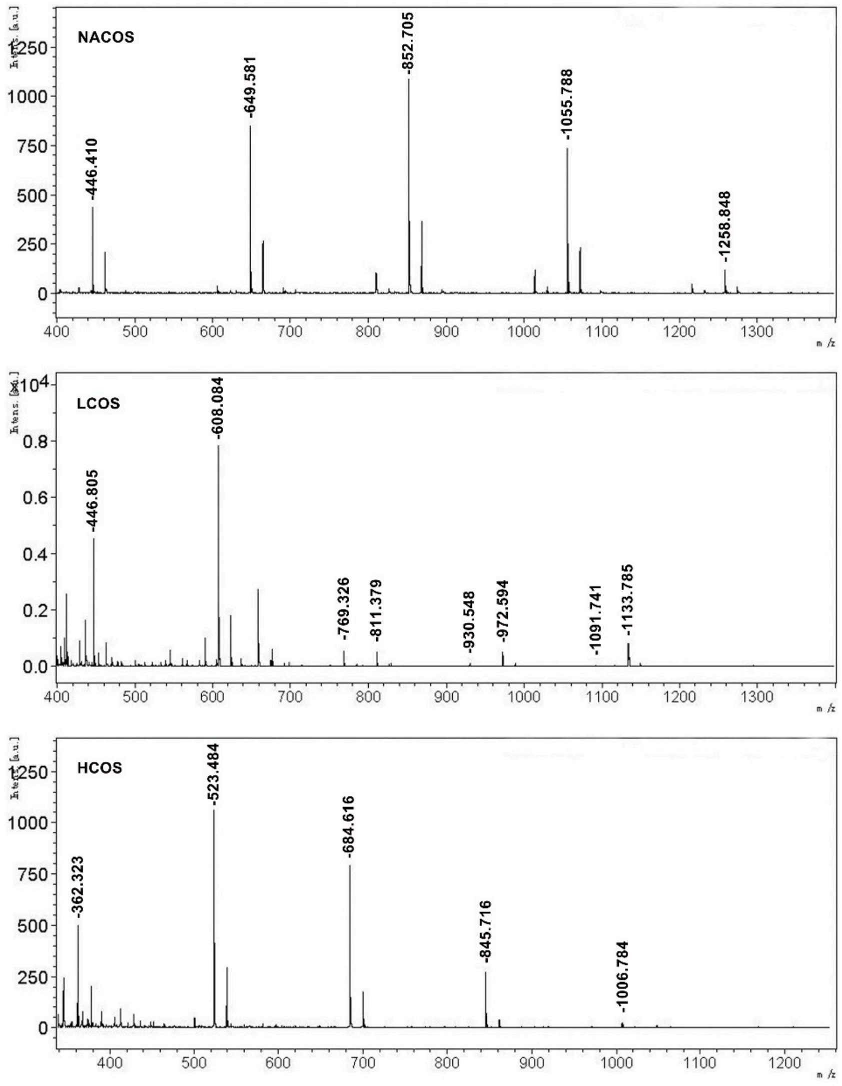

Fig. 3. The average molecular weights were below 1 kDa by HPLC using ELSD detection on a TSKgel G4000PWXL (Tosoh corporation) column (7.8 mm× 300 mm), with 1 k, 5 k and 25 kDa dextran as standards.

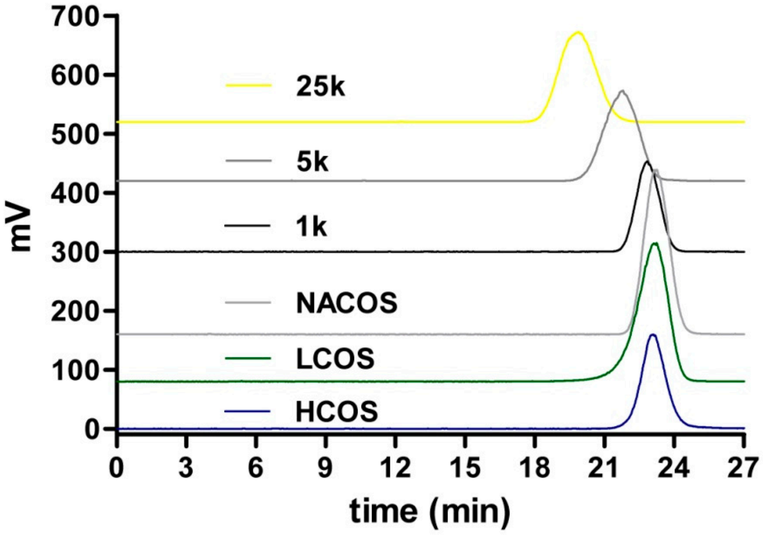

Fig. 4. Determination of the deacetylation degree (DD) of COSs by ^{1}H-NMR spectroscopy. The DD was calculated using the peak integration of proton H2-6 of both monomers at 3.0-4.0 ppm and three protons of the acetyl group at 2.1 ppm.

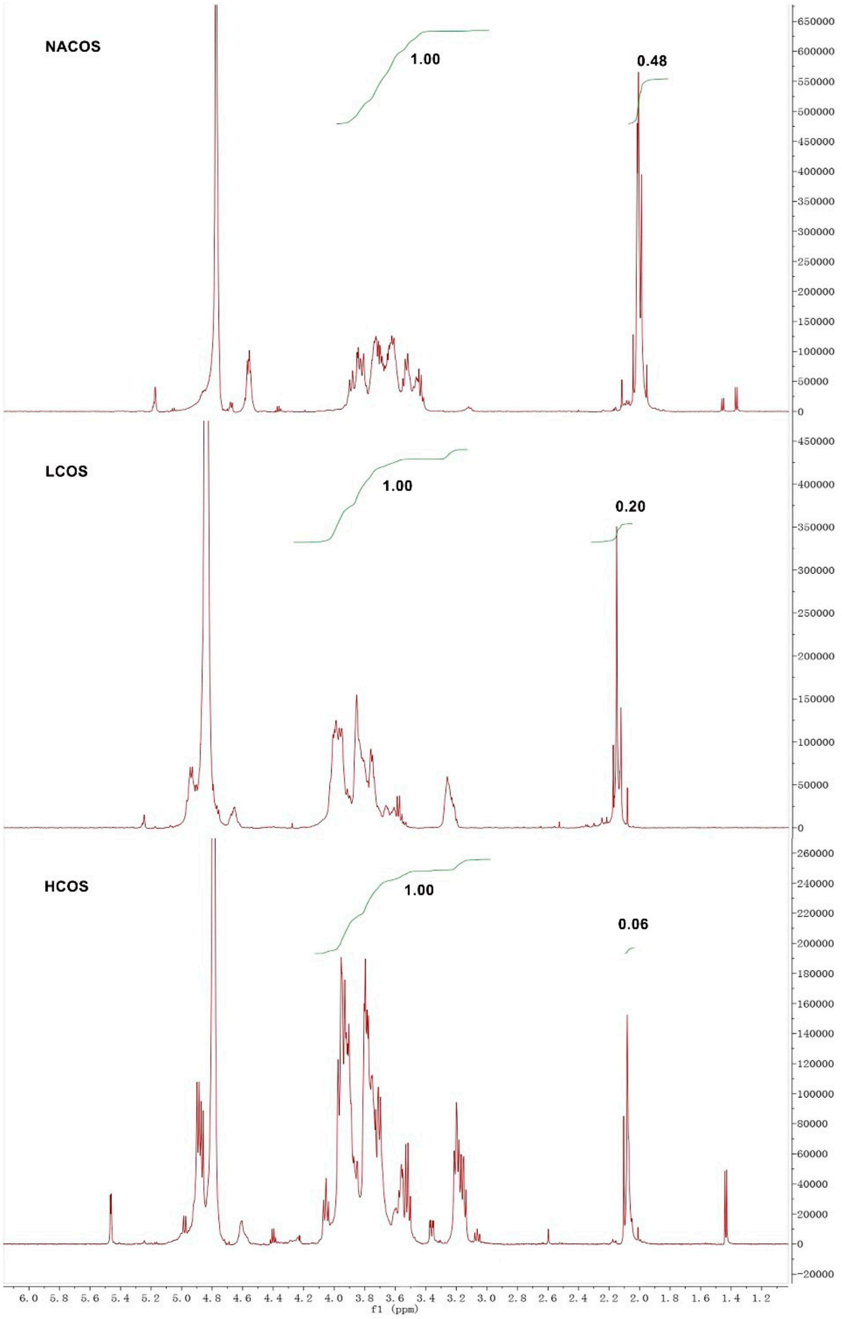

Fig. 5. Effect of COS conjugation on CD3$^+$CD4$^+$ T cell population from mouse spleens by flow cytometry analysis. On day 14 after the third immunization, mice were euthanized and splenic lymphocytes were prepared. The CD3$^+$CD4$^+$ T cell population was determined by flow cytometry analysis. Numbers in each quadrant show percentages of CD3$^-$CD4$^+$ (Upper Left), CD3$^+$CD4$^+$ (Upper Right), CD3$^+$CD4$^-$ (Lower Left), CD3$^-$CD4$^-$ (Lower Right), stained with APC-conjugated monoclonal antibody against CD3 and FITC-labelled monoclonal antibody against CD4.

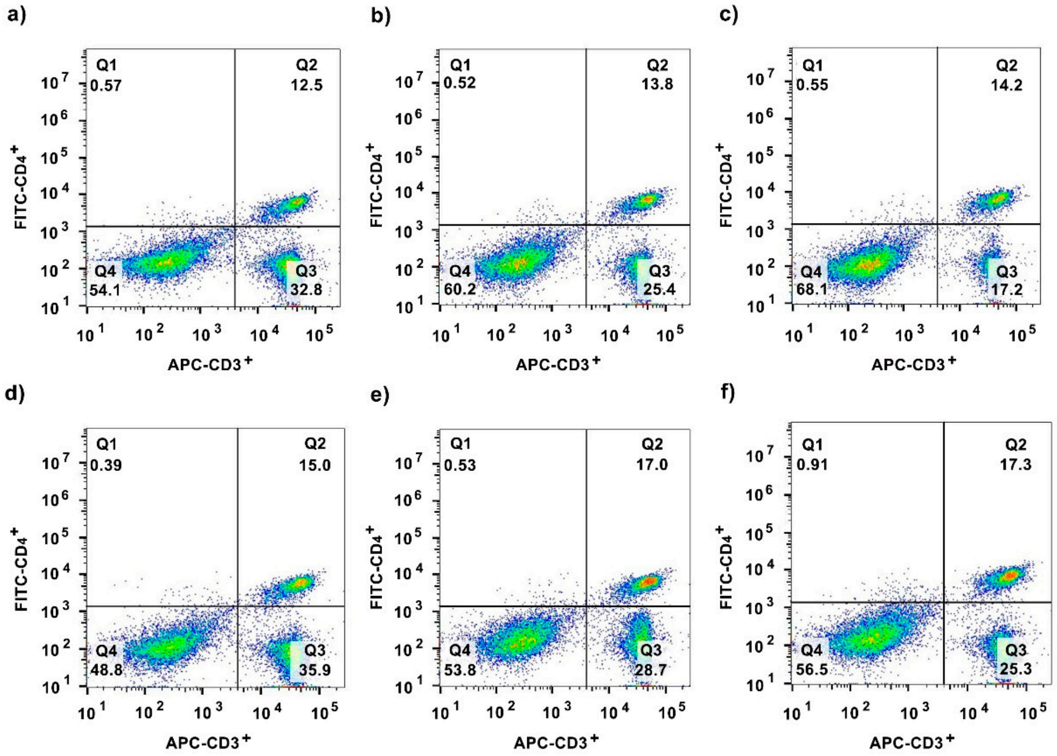

Fig. 6. Effect of COS conjugation on CD3$^+$CD8$^+$ T cell population from mouse spleens by flow cytometry analysis. On day 14 after the third immunization, mice were euthanized and splenic lymphocytes were prepared. And the CD3$^+$CD8$^+$ T cell population was determined by flow cytometry analysis. Numbers in each quadrant show percentages ofCD3$^-$CD8$^+$ (Upper Left), CD3$^+$CD8$^+$ (Upper Right), CD3$^+$CD8$^-$ (Lower Left), CD3$^-$CD8$^-$ (Lower Right), stained with APC-conjugated monoclonal antibody against CD3 and PE-labelled monoclonal antibody against CD8.

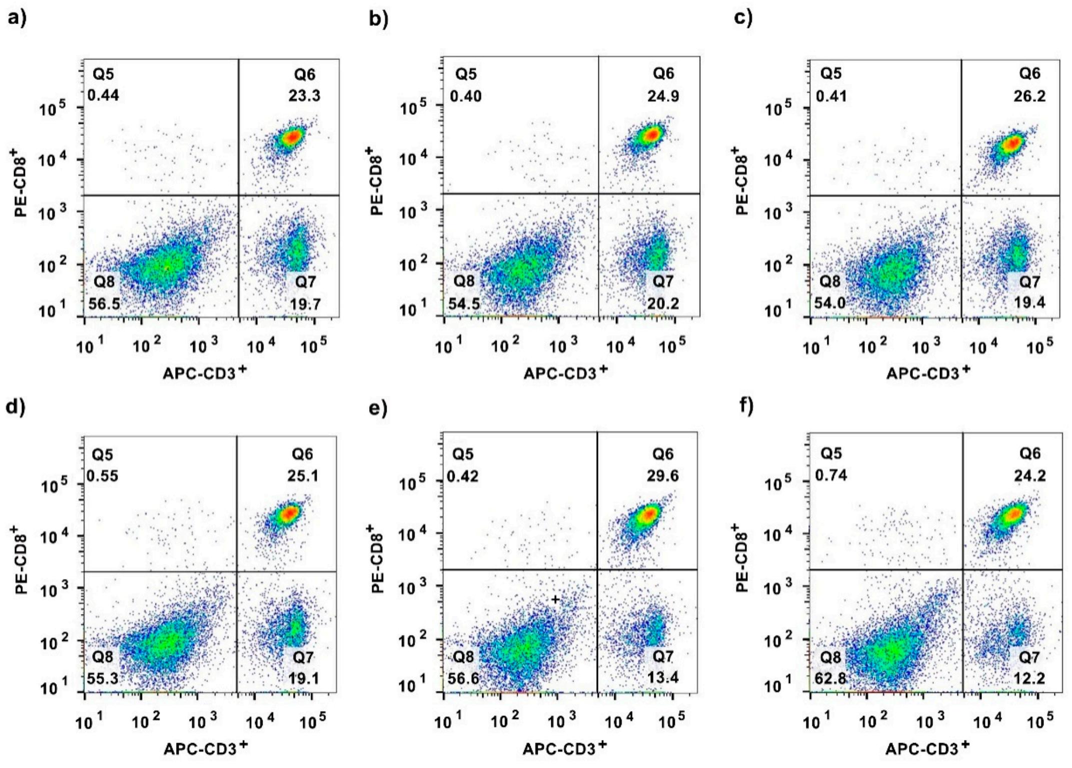

Tables

Table 1. Effect of COS conjugation on the ratio of PCV2-specific IgG2a to IgG1 in mouse serum after immunization

Groups	14 dpi	28 dpi	42 dpi
PBS	0.60±0.06	0.63±0.08	0.66±0.05
PCV2	0.48±0.05	0.46±0.02	0.48±0.03
NACOS-PCV2	0.43±0.05*	0.53±0.04*	0.55±0.06*
LCOS-PCV2	0.52±0.04	0.47±0.05	0.57±0.04*
HCOS-PCV2	0.47±0.04	0.50±0.05*	0.64±0.05**
ISA206/PCV2	0.55±0.08*	0.52±0.08	0.56±0.09

The IgG2a/IgG1 ratio for each mouse serum was calculated and representative results for experimental groups (n=6) were presented as means ± SD. *$P<0.05$ or **$P<0.01$, compared to the PCV2 alone group. dpi: days post primary immunization
